# Hexakis(dimethyl sulfoxide-κ*O*)chromium(III) trichloride

**DOI:** 10.1107/S1600536808016784

**Published:** 2008-06-13

**Authors:** Yuliya M. Mikhaylichenko, Matti Haukka, Vadim O. Pavlenko, Igor O. Fritsky, Turganbay S. Iskenderov

**Affiliations:** a National Taras Shevchenko University, Department of Chemistry, Volodymyrska Street 64, 01601 Kiev, Ukraine; b Department of Chemistry, University of Joensuu, PO Box 111, 80101 Joensuu, Finland; c Department of Chemistry, Karakalpakian University, Universitet Keshesi 1, 742012 Nukus, Uzbekistan

## Abstract

In the title compound, [Cr(C_2_H_6_OS)_6_]Cl_3_, each Cr^III^ ion is located on a three-fold inversion axis and is coordinated by six dimethyl­sulfoxide ligands [Cr—O = 1.970 (2)–1.972 (2) Å; O—Cr—O = 88.19 (9) and 91.81 (9)°] in a slightly distorted octa­hedral geometry. The Cl^−^ anions take part in the formation of weak C—H⋯Cl hydrogen bonds, which contribute to the crystal packing stabilization.

## Related literature

For related literature, see: Chan *et al.* (2004 ▶); Desiraju & Steiner (1999 ▶); Öhrström & Svensson (2000 ▶); Persson *et al.* (1995 ▶, and references therein); Reynolds (1970 ▶).
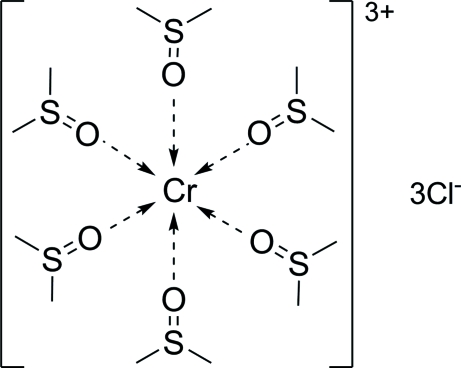


## Experimental

### 

#### Crystal data

Cr(C_2_H_6_OS)_6_]Cl_3_
*M* *_r_* = 627.12
Trigonal, 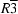

*a* = 10.5499 (6) Å
*c* = 21.1370 (13) Å
*V* = 2037.4 (2) Å^3^
*Z* = 3
Mo *K*α radiation
μ = 1.20 mm^−1^
*T* = 120 (2) K
0.34 × 0.29 × 0.20 mm

#### Data collection

Nonius KappaCCD diffractometer
Absorption correction: multi-scan (North *et al.*, 1968 ▶) *T*_min_ = 0.688, *T*_max_ = 0.795
10865 measured reflections
1044 independent reflections
855 reflections with *I* > 2σ(*I*)
*R*_int_ = 0.051

#### Refinement

*R*[*F* ^2^ > 2σ(*F* ^2^)] = 0.042
*wR*(*F* ^2^) = 0.135
*S* = 1.14
1044 reflections
46 parameters
H-atom parameters constrained
Δρ_max_ = 0.82 e Å^−3^
Δρ_min_ = −0.48 e Å^−3^

### 

Data collection: *APEX2* (Bruker, 2004 ▶); cell refinement: *DENZO*/*SCALEPACK* (Otwinowski & Minor, 1997 ▶); data reduction: *DENZO*/*SCALEPACK*; program(s) used to solve structure: *SIR2004* (Burla *et al.*, 2005 ▶); program(s) used to refine structure: *SHELXL97* (Sheldrick, 2008 ▶); molecular graphics: *ORTEP-3 for Windows* (Farrugia, 1997 ▶); software used to prepare material for publication: *SHELXL97*.

## Supplementary Material

Crystal structure: contains datablocks I, global. DOI: 10.1107/S1600536808016784/cv2410sup1.cif

Structure factors: contains datablocks I. DOI: 10.1107/S1600536808016784/cv2410Isup2.hkl

Additional supplementary materials:  crystallographic information; 3D view; checkCIF report

## Figures and Tables

**Table 1:** Hydrogen-bond geometry (Å, °)

*D*—H⋯*A*	*D*—H	H⋯*A*	*D*⋯*A*	*D*—H⋯*A*
C1—H1*B*⋯Cl1	0.98	2.75	3.647 (3)	153
C1—H1*A*⋯Cl2^i^	0.98	2.64	3.614 (4)	176

Symmetry code: (i) 
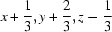
.

**Fig. 1.**
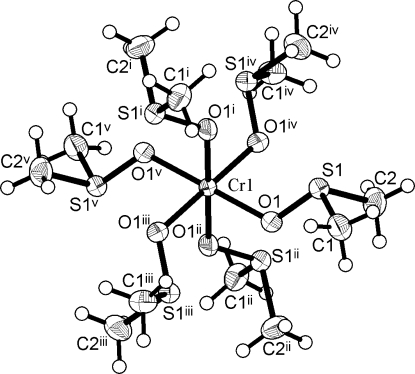
The molecular structure of (I), showing the atom-numbering scheme and displacement ellipsoids at the 60% probability level [symmetry codes: (i) -x + y, 1 - x, z; (ii) 2/3 + x-y, 1/3 + x, 1/3 - z; (iii) 1 - y, 1 + x-y, z; (iv) -1/3 + y, 1/3 - x + y, 1/3 - z; (v) 2/3 - x, 4/3 - y, 1/3 - z.]

**Fig. 2.**
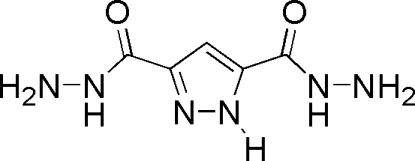
1H-pyrazole-3,5-dicarbohydrazide

## supplementary crystallographic information

### Comment

Dimethylsulfoxide (dmso) has often been used as solvent and a ligand in inorganic chemistry since the beginning of the 1960th. Dimethylsulfoxide is a monodentate O–,*S*-donor ligand (Reynolds, 1970). Solvates of some transition metal ions have been prepared and structurally charaterized (Persson *et al.*, 1995 and references therein).

The title compound, (I), is composed of [Cr(C_2_H_6_OS)_6_]^3+^ cations and chloride anions. The Cr(III) ion is located on a 3-fold inversion axis being coordinated by the six dimethylsulfoxide ligands in a slightly distorted octahedral geometry (Fig. 1), with Cr—O 1.970 (2)–1.972 (2) Å interatomic distances and O—Cr—O 88.19 (9), 91.81 (9)° bond angles. A search in the Cambridge Structural Database revealed 14 reports of compounds containing transition metal hexakis(dimethylsulfoxide) cations, of which two described the structure of the [Cr(C_2_H_6_OS)_6_]^3+^ cation in [Cr(C_2_H_6_OS)_6_](ClO_4_)_3_ (Chan *et al.*, 2004) and [Cr(C_2_H_6_OS)_6_](NO_3_)_3_ (Ohrstrom & Svensson, 2000). The S═O bond lengths in the aforementioned compounds are almost identical, 1.542 (3) Å *versus* 1.543 (2) Å for the title compound, as well as the O—Cr—O angles, 87.9/92.2° *versus* 88.19–91.81°. In the present structure the average value of Cr—O—S angles (121.9°) is somewhat smaller than that in [Cr(C_2_H_6_OS)_6_](NO_3_)_3_ (123.6°). All other angles and bonds of the title compound are very similar to the above mentioned structures.

In (I), the Cl anions take part in formation of weak C—H···Cl hydrogen bonds (Table 1), which contribute to the crystal packing stabilization.

### Experimental

Complex (I) was synthesized during the attempt to prepare chromium (III) complex with 1*H*-pyrazole-3,5-dicarbohydrazide (Fig. 2) by adding CrCl_3_.6H_2_O (0.3 mmol, 3 ml of 0.1*M* aqueous solution) to the 1*H*-pyrazole-3,5-dicarbohydrazide (0.0552 g, 0.3 mmol) in dimethylsulfoxide solution (6 ml). The mixture was stirred for 30 min at ambient temperature. The resulting green solution was filtered and the filtrate was left to stand at room temperature. Slow evaporation of the solvent during 2 weeks yielded green crystals of (I).

### Refinement

The H atoms were positioned geometrically (C—H 0.98 Å) and allowed to ride on their parent atoms, with U_iso_(H) = 1.5U_eq_(C).

### Crystal data

**Table:**

[Cr(C_2_H_6_OS)_6_]Cl_3_	*Z* = 3
*M**_r_* = 627.12	*F*_000_ = 981
Trigonal, *R*3	*D*_x_ = 1.533 Mg m^−^^3^
Hall symbol: -R 3	Mo *K*α radiation λ = 0.71073 Å
*a* = 10.5499 (6) Å	Cell parameters from 6073 reflections
*b* = 10.5499 (6) Å	θ = 1.0–27.5º
*c* = 21.1370 (13) Å	µ = 1.20 mm^−^^1^
α = 90º	*T* = 120 (2) K
β = 90º	Block, green
γ = 120º	0.34 × 0.29 × 0.20 mm
*V* = 2037.4 (2) Å^3^	

### Data collection

**Table:**

Nonius KappaCCD diffractometer	1044 independent reflections
Radiation source: fine-focus sealed tube	855 reflections with *I* > 2σ(*I*)
Monochromator: horizontally mounted graphite crystal	*R*_int_ = 0.051
Detector resolution: 9 pixels mm^-1^	θ_max_ = 27.6º
*T* = 120(2) K	θ_min_ = 2.4º
φ scans and ω scans with κ offset	*h* = −13→13
Absorption correction: multi-scan(North *et al.*, 1968)	*k* = −13→13
*T*_min_ = 0.688, *T*_max_ = 0.795	*l* = −26→27
10865 measured reflections	

### Refinement

**Table:**

Refinement on *F*^2^	Secondary atom site location: difference Fourier map
Least-squares matrix: full	Hydrogen site location: inferred from neighbouring sites
*R*[*F*^2^ > 2σ(*F*^2^)] = 0.042	H-atom parameters constrained
*wR*(*F*^2^) = 0.135	*w* = 1/[σ^2^(*F*_o_^2^) + (0.0789*P*)^2^ + 3.3338*P*] where *P* = (*F*_o_^2^ + 2*F*_c_^2^)/3
*S* = 1.14	(Δ/σ)_max_ < 0.001
1044 reflections	Δρ_max_ = 0.82 e Å^−^^3^
46 parameters	Δρ_min_ = −0.48 e Å^−^^3^
Primary atom site location: structure-invariant direct methods	Extinction correction: none

### Special details

**Table:**

Geometry. All e.s.d.'s (except the e.s.d. in the dihedral angle between two l.s. planes) are estimated using the full covariance matrix. The cell e.s.d.'s are taken into account individually in the estimation of e.s.d.'s in distances, angles and torsion angles; correlations between e.s.d.'s in cell parameters are only used when they are defined by crystal symmetry. An approximate (isotropic) treatment of cell e.s.d.'s is used for estimating e.s.d.'s involving l.s. planes.
Refinement. Refinement of *F*^2^ against ALL reflections. The weighted *R*-factor *wR* and goodness of fit *S* are based on *F*^2^, conventional *R*-factors *R* are based on *F*, with *F* set to zero for negative *F*^2^. The threshold expression of *F*^2^ > σ(*F*^2^) is used only for calculating *R*-factors(gt) *etc*. and is not relevant to the choice of reflections for refinement. *R*-factors based on *F*^2^ are statistically about twice as large as those based on *F*, and *R*- factors based on ALL data will be even larger.

### Fractional atomic coordinates and isotropic or equivalent isotropic displacement parameters (Å^2^)

**Table:**

	*x*	*y*	*z*	*U*_iso_*/*U*_eq_	
Cr1	0.3333	0.6667	0.1667	0.0214 (3)	
Cl1	0.0000	0.0000	0.0000	0.0324 (5)	
Cl2	0.0000	0.0000	0.25064 (7)	0.0416 (4)	
S1	0.24193 (8)	0.38772 (8)	0.08860 (4)	0.0261 (3)	
O1	0.3687 (2)	0.5325 (2)	0.11455 (10)	0.0270 (5)	
C1	0.3083 (4)	0.3721 (4)	0.01371 (16)	0.0336 (7)	
H1A	0.3141	0.4485	−0.0145	0.050*	
H1B	0.2416	0.2757	−0.0044	0.050*	
H1C	0.4058	0.3834	0.0186	0.050*	
C2	0.2577 (4)	0.2497 (4)	0.12936 (18)	0.0384 (8)	
H2A	0.3576	0.2670	0.1248	0.058*	
H2B	0.1883	0.1538	0.1116	0.058*	
H2C	0.2360	0.2518	0.1743	0.058*	

### Atomic displacement parameters (Å^2^)

**Table:**

	*U*^11^	*U*^22^	*U*^33^	*U*^12^	*U*^13^	*U*^23^
Cr1	0.0165 (4)	0.0165 (4)	0.0311 (6)	0.00824 (19)	0.000	0.000
Cl1	0.0245 (6)	0.0245 (6)	0.0480 (11)	0.0123 (3)	0.000	0.000
Cl2	0.0421 (6)	0.0421 (6)	0.0405 (9)	0.0211 (3)	0.000	0.000
S1	0.0221 (4)	0.0207 (4)	0.0346 (5)	0.0099 (3)	−0.0004 (3)	−0.0004 (3)
O1	0.0202 (10)	0.0222 (10)	0.0374 (12)	0.0096 (9)	−0.0015 (8)	−0.0045 (8)
C1	0.0339 (17)	0.0279 (16)	0.0331 (17)	0.0111 (13)	0.0028 (13)	−0.0017 (13)
C2	0.045 (2)	0.0278 (16)	0.044 (2)	0.0196 (15)	−0.0007 (16)	0.0063 (14)

### Geometric parameters (Å, °)

**Table:**

Cr1—O1^i^	1.972 (2)	S1—C1	1.772 (3)
Cr1—O1^ii^	1.971 (2)	C1—H1A	0.9800
Cr1—O1^iii^	1.971 (2)	C1—H1B	0.9800
Cr1—O1	1.970 (2)	C1—H1C	0.9800
Cr1—O1^iv^	1.971 (2)	C2—H2A	0.9800
Cr1—O1^v^	1.971 (2)	C2—H2B	0.9800
S1—O1	1.542 (2)	C2—H2C	0.9800
S1—C2	1.770 (3)		
			
O1^i^—Cr1—O1^ii^	180.0	O1—S1—C1	102.86 (14)
O1^i^—Cr1—O1^iii^	91.81 (9)	C2—S1—C1	98.87 (17)
O1^ii^—Cr1—O1^iii^	88.19 (9)	S1—O1—Cr1	121.86 (12)
O1^i^—Cr1—O1	91.81 (9)	S1—C1—H1A	109.5
O1^ii^—Cr1—O1	88.19 (9)	S1—C1—H1B	109.5
O1^iii^—Cr1—O1	91.81 (9)	H1A—C1—H1B	109.5
O1^i^—Cr1—O1^iv^	88.19 (9)	S1—C1—H1C	109.5
O1^ii^—Cr1—O1^iv^	91.81 (9)	H1A—C1—H1C	109.5
O1^iii^—Cr1—O1^iv^	180.0	H1B—C1—H1C	109.5
O1—Cr1—O1^iv^	88.19 (9)	S1—C2—H2A	109.5
O1^i^—Cr1—O1^v^	88.19 (9)	S1—C2—H2B	109.5
O1^ii^—Cr1—O1^v^	91.81 (9)	H2A—C2—H2B	109.5
O1^iii^—Cr1—O1^v^	88.19 (9)	S1—C2—H2C	109.5
O1—Cr1—O1^v^	179.999 (1)	H2A—C2—H2C	109.5
O1^iv^—Cr1—O1^v^	91.81 (9)	H2B—C2—H2C	109.5
O1—S1—C2	104.46 (15)		
			
C2—S1—O1—Cr1	−112.64 (17)	O1^ii^—Cr1—O1—S1	140.87 (18)
C1—S1—O1—Cr1	144.52 (16)	O1^iii^—Cr1—O1—S1	−131.00 (10)
O1^i^—Cr1—O1—S1	−39.13 (18)	O1^iv^—Cr1—O1—S1	49.00 (10)

Symmetry codes: (i) −*x*+*y*, −*x*+1, *z*; (ii) *x*−*y*+2/3, *x*+1/3, −*z*+1/3; (iii) −*y*+1, *x*−*y*+1, *z*; (iv) *y*−1/3, −*x*+*y*+1/3, −*z*+1/3; (v) −*x*+2/3, −*y*+4/3, −*z*+1/3.

### Hydrogen-bond geometry (Å, °)

**Table:**

*D*—H···*A*	*D*—H	H···*A*	*D*···*A*	*D*—H···*A*
C1—H1B···Cl1	0.98	2.75	3.647 (3)	153
C1—H1A···Cl2^vi^	0.98	2.64	3.614 (4)	176

Symmetry codes: (vi) *x*+1/3, *y*+2/3, *z*−1/3.
